# Supplementary material for: Strain-engineered inverse charge-funnelling in layered semiconductors
Source: Nat Commun. 2018 Apr 25;9:1652. doi: 10.1038/s41467-018-04099-7 (PMC5916941; doi:10.1038/s41467-018-04099-7)
Supplement: Supplementary file 1 — Supplementary Information [file 41467_2018_4099_MOESM1_ESM.pdf]

SUPPLEMENTARY MATERIAL
Strain-engineered inverse charge-funnelling in layered semiconductors

De Sanctis et al.

Supplementary Notes

Supplementary Note 1 Photo-oxidation mechanism

Following the model in ref. 1, we propose in Supplementary Figure 1a the energy diagram of the interface between HfS_2 and aqueous oxygen in atmosphere[2] provided by the reaction $2H_2O \rightleftharpoons O_2(aq) + 4e^- + 4H^+$. The intrinsic chemical potential of HfS_2 is $\mu_i = -5.2\,\text{eV}$ (calculated from the distance from the vacuum level to the top of the valence band, $\phi = -6.68\,\text{eV}$ and indirect bandgap $E_g = 1.96\,\text{eV}$), while that of the oxygen acceptor state is $\mu^0_{\text{redox}} = -4.1\,\text{eV}$. This induces a band-bending at the surface of the layered semiconductor such that an optical transition above the bandgap can make an electron ready to be transferred to the oxygen empty states, making the photo-oxidation reaction highly feasible across the whole visible range, explaining the high instability of few-layer HfS_2 in atmospheric conditions. The CT reaction starts with a photon of energy $h\nu$ impinging on HfS_2, which produces an optical excitation, leaving the material in an excited state: $HfS_2 + h\nu \longrightarrow {HfS_2}^*$. This state provides the carriers for the CT reaction at the surface according to the following:

$$ {HfS_2}^* + O_2(aq) \longrightarrow HfS_2 + {O_2}^{\cdot -}(aq) + h^+, \tag{1} $$

the oxygen radical ion ${O_2}^{\cdot -}(aq)$ can then react with the HfS_2 and, upon cleavage of the Hf−S bond, bind to the Hf and S, respectively:

$$ Hf + 2S + 3{O_2}^{\cdot -}(aq) + 3h^+ \longrightarrow HfO_2 + 2\,SO_2(g). \tag{2} $$

Therefore the total reaction can be written as:

$$ HfS_2(s) + 3O_2(aq) + h\nu \longrightarrow HfO_2(s) + 2SO_2(g). \tag{3} $$

Marcus-Gerischer theory (MGT)[2] gives an estimation of the rate of change in the pristine material composition:

$$ \frac{d\Theta}{dt} \propto \Theta \Phi_{\text{ph}}[O_2] \exp\left[-\frac{\left(\frac{E_g}{2} + E_i - E^0_{\text{F,red}} - \lambda\right)^2}{4k_b T \lambda} \right], \tag{4} $$

where Θ is the amount of pristine material, Φ_{ph} is the laser flux, $[O_2]$ is the oxygen concentration, E_g is the direct gap energy, E_i is the intrinsic Fermi level, $E^0_{\text{F,red}} \sim 3.1\,\text{eV}$ is the energy of the oxygen acceptor state with respect to the vacuum level, λ is the renormalization energy of oxygen in water ($\sim 1\,\text{eV}$), k_b is the Boltzmann constant and T the temperature[2]. Therefore, the oxidation rate depends on the laser flux Φ_{ph} and the initial amount of pristine material Θ_0: $\Theta \propto \Theta_0 \exp(-t/\tau)$ where the decay time $\tau \propto \Phi^{-1}_{\text{ph}}$. This dependence is experimentally verified in Supplementary Figure 1b-c where the intensity of the Raman A_{1g} mode of HfS_2, an indication of the amount of pristine material present in the sampling volume, is plotted against time for different laser fluxes. Monoexponential decay fits give a decay time τ with the expected power relation (Supplementary Figure 1c, inset).

Supplementary Figure 1d shows that the oxidized area is compatible with the diffraction-limited spot size of our laser system (see methods). Indeed, the AFM topography and tapping phase images of an exposed thick (80 nm) flake shows a bubble-like structure with a 25 % increase in height. This feature shows a Gaussian profile with a full-width at half-maximum (FWHM) of 260 nm. We then exposed different areas of the flake, for different lengths of time while keeping the laser power density constant. As shown in Supplementary Figure 1e, no changes in the FWHM of the Gaussian profile is observed. All these observations confirm that photon-assisted oxidation is taking place, as opposed to thermal oxidation.

Supplementary Note 2 Laser-induced oxidation: AFM data

In Supplementary Figure 2 we report additional data on the laser-induced oxidation process in HfS_2. Supplementary Figure 2a-d show the AFM data acquired on a flake with different layers deposited on a Si/SiO_2 substrate (oxide thickness 285 nm). Different areas of the flake were exposed with $\lambda_{exc} = 532$nm at a power of $0.1\,\mathrm{MW\,cm^{-2}}$ for 20 s, using a commercial *Renishaw* micro-Raman spectroscopy system. A marked change of contrast in the areas of HfS_2 exposed to the laser is visible in the white light optical micrograph (see Supplementary Figure 2a). Though the observed change of contrast might suggest that HfS_2 has been ablated by the laser, a detailed study of the topography of the flakes with atomic force microscopy demonstrates that this is not the case, see Supplementary Figure 2b. Indeed, we measured that the average height of the laser-exposed area is the same as the nearby control area (Supplementary Figure 2d), $< \mathrm{h} > \simeq 8\,\mathrm{nm}$, while the root-mean-square (rms) height of the surface (roughness), S_q, is increased by $\sim 50\,\%$ with respect to the control areas (which have an S_q value comparable with that of the underlying substrate).

Supplementary Figure 2e-g show additional AFM data for the flake of Figure 1 in the main text. The AFM topography and tapping phase images are shown in panels e and f, respectively. Panel g shows the height profiles for the two different regions of the flake. The height of one layer of HfS_2 was measured to be $0.76 \pm 0.08\,\mathrm{nm}$ (panel c), thus the aforementioned flake has a thickness of ~ 20 and ~ 14 layers in the two regions across which the laser was scanned.

In Supplementary Figure 2h we report the Raman spectra of HfS_2 on a Si/SiO_2 substrate, shown in the optical micrograph inset. In the bulk material we observe all the first order Raman modes: $A_{1g} \simeq 337\,\mathrm{cm^{-1}}$, $E_g \simeq 260\,\mathrm{cm^{-1}}$, $E_{u(LO)} \simeq 321\,\mathrm{cm^{-1}}$ and $E_{u(TO)} \simeq 136\,\mathrm{cm^{-1}}$; All these modes are well described by a fit with a Lorentzian curve with a full-width-at-half-maximum (FWHM) $\sim 10\,\mathrm{cm^{-1}}$ and the peak positions agree well with previous studies[3, 4, 5], except for the $E_{u(TO)}$ mode which appears blue-shifted (literature value $\sim 155-166\,\mathrm{cm^{-1}}$). For thinner flakes we observe that it is increasingly difficult to acquire a spectrum as the material is thinned. To the best of our knowledge no reports have shown the isolation of single-layer HfS_2.

Supplementary Note 3 Photon assisted oxidation of bulk HfS_2

Supplementary Figure 3 shows the photo-oxidation of a thick (230 nm) HfS_2 flake. A comparison of micrograph pictures before (Supplementary Figure 3a) and after (Supplementary Figure 3b) exposing with a laser a 5×5 μm square area ($\lambda_{exc} = 375$ nm, $P = 5.0$ MW/cm^2, 0.5 μm steps each exposed for 20 s) shows that for bulk flakes the oxidation does not involve the whole thickness, as it is not possible to see the substrate underneath the flake. Indeed, AFM topography (Supplementary Figure 3c) and tapping phase (Supplementary Figure 3d) show that the surface of the flake is damaged after prolonged exposure, as shown by the line profiles across the laser-exposed region.

Supplementary Note 4 Supplementary AFM data

The data regarding the absorption coefficient, presented in Figure 4 in the main text and in Supplementary Figure 5, have been acquired on a 10.5 nm thick flake, as shown in Supplementary Figure 4a. The data shown in Figure 2c in the main text have been acquired on a uniform 4.9 nm thick flake, as shown in Supplementary Figure 4b.

Supplementary Note 5 Visible wavelength absorption and photoluminescence extended data

Supplementary Figure 5a shows the absorption coefficient of pristine HfS_2 as acquired on a thin flake in our experimental setup (see methods). The data are in good agreement with the literature[6]. Transmittance (T), reflectance and absorbance (1-T-R) are shown in Supplementary Figure 5b. Supplementary Figure 5c shows the photoluminescence (PL) spectra of two representative flakes of bulk HfS_2 under 473 nm excitation. No strong exciton peak can be seen in proximity of the indirect gap energy whilst the feature at $\sim 1.7\,\text{eV}$ has been attributed to impurity states in the gap[7].

Supplementary Note 6 Reactions energy cost

The feasibility of the reaction in Supplementary Equation (3) has been verified by simulating the crystal structure of the different compounds and calculating the energy cost per reaction, as shown in Supplementary Figure 6 and Supplementary Table 1. The energy cost was calculated as $E_\text{R} = E_\text{reagents} - E_\text{products}$.

We also considered the possibility of the formation of two-dimensional (2D) HfO_2, as opposed to monoclinic 3D HfO_2. Such material would be given by the substitution of each S by O in the 2D precursor. However, our calculation shows that the proposed reaction in Supplementary Equation (3) has an energy cost of $-10.90\,\text{eV}$ for 2D HfO_2, a difference $\Delta E = 0.68\,\text{eV}$ as compared to 3D (monoclinic) HfO_2, which makes the formation of 2D HfO_2 not favourable.

Supplementary Note 7 HfS_2 and HfO_2 band structure

In Supplementary Figure 7a,b we report the calculated band structures of monoclinic HfO_2 and HfS_2, respectively. In the latter the indirect band gap is $\sim$ 1.38 eV in the $\Gamma \rightarrow$ M direction, while the direct gap at the Γ point is $\sim$ 2.1 eV. These values are $\sim$ 40% off the measured ones[6, 8] and in line with other reported DFT calculations[9]. Supplementary Figure 7c shows the calculated conduction band minimum (CBm) and valence band maximum (VBM) at Γ and in the $\Gamma \rightarrow$ M direction, as a function of strain. Supplementary Figure 7d shows the energy difference between the strained and unstrained VBM and CBm.

Supplementary Note 8 Electrical characterization

Supplementary Figure 8 shows the electrical characterization of the device presented in Figure 3 in the main text. Supplementary Figure 8a shows the current-voltage (I_sd-V_sd) characteristic before the photo-oxidation at different incident optical powers and Supplementary Figure 8b shows the same after the photo-oxidation. Sweeping the V_sd in both direction, no significant hysteresis is observed. In Supplementary Supplementary Figure 8c we show a gate voltage sweep as a function of incident optical power for the same device. In this case a very large hysteresis is observed, due to the ambient contamination of the device since it is measured in air at room temperature. The observed hysteresis is in agreement with previous results[10].

Supplementary Note 9 Extended photocurrent mapping data

Supplementary Figure 9a shows the scanning photocurrent microscopy (SPCM) map of the device presented in Figure 4 of the main text, prior to laser-assisted oxidation. Under a bias of $V_\text{sd} = \pm 1\,\text{V}$, the map shows a very small photocurrent generated in the flake, which

does not change with the sign of the bias. Therefore we attribute this small contribution to impurities on the surface causing local doping of the material. No measurable photocurrent was recorded at zero bias. Supplementary Figure 9b-c show the SPCM maps of another device before and after laser assisted oxidation in the centre of the flake, under $V_{\mathrm{sd}} = 5\,\mathrm{V}$. This device shows the same behaviour as the one presented in Figure 3, main text, where a strong localized photocurrent is observed after laser-assisted oxidation (green circle). Before laser oxidation no significant photocurrent can be observed, as in the previous case.

Supplementary Note 10 Analytical model describing the observed SPCM response

In this section we develop a simple analytical model which allows to simulate the scanning photocurrent microscopy (SPCM) results shown in the main text (see Figure 4f, main text).

In order to describe how local illumination results in a spatial map of the photoresponse of a device we need to consider the processes of generation, motion and collection of charges. Starting from the charge continuity equation:

$$\frac{1}{q}\frac{\partial \rho}{\partial t} = G - R - \frac{1}{q}\boldsymbol{\nabla}\cdot\mathbf{j}, \tag{5}$$

where ρ is the charge density, G and R are the generation and recombination rates of the carriers, respectively and $\mathbf{j}$ is the current density. Assuming that the average carriers diffusion length is larger than the laser-spot size we can take G to be a delta function centred at the laser excitation point. The recombination rate is equal to $R_{\mathrm{i}} = -\Delta n_{\mathrm{i}}/\tau_{\mathrm{i}}$, where $\mathrm{i} = \mathrm{e}, \mathrm{h}$ indicates the two types of carrier, τ is the carrier recombination lifetime and $\Delta n_{\mathrm{i}} = n_{\mathrm{i}}^{\mathrm{t}} - n_{\mathrm{i}}^{\mathrm{d}}$ is the excited carriers density (difference between the total carrier density $n_{\mathrm{i}}^{\mathrm{t}}$ and the carrier density in absence of illumination $n_{\mathrm{i}}^{\mathrm{d}}$). The carrier density can be decomposed into drift and diffusion terms (we neglect thermoelectric effects):

$$\mathbf{j} = q(n_{\mathrm{e}}\mu_{\mathrm{e}} - n_{\mathrm{h}}\mu_{\mathrm{h}})\mathbf{E} + q(D_{\mathrm{e}}\boldsymbol{\nabla} n_{\mathrm{e}} - D_{\mathrm{h}}\boldsymbol{\nabla} n_{\mathrm{h}}), \tag{6}$$

where μ_{i} is the mobility, $\mathbf{E}$ is the electric field across the device and $D_i = (k_{\mathrm{b}}T\mu_{\mathrm{i}})/q$ is the diffusion coefficient at temperature T with $k_{\mathrm{b}} \simeq 8.617 \cdot 10^{-5}\,\mathrm{eV/K}$ the Boltzmann constant. In a junction device or in the presence of a Schottky contact the photocurrent is dominated by the minority carriers since the drift and diffusion components of the majority carriers will cancel each other[11]. It is therefore possible to recast Supplementary Equation (5) in terms of one type of carrier:

$$\frac{\partial n_{\mathrm{e}}}{\partial t} = -\frac{\Delta n_{\mathrm{e}}}{\tau_{\mathrm{e}}} - \frac{1}{q}\boldsymbol{\nabla}\cdot\mathbf{j}_{\mathrm{e}}. \tag{7}$$

It is important to notice the spatial dependence of Δn_{e} and $\mathbf{E}$. Therefore, for a 1D system, Supplementary Equation (7) takes the form:

$$\frac{\partial n_{\mathrm{e}}}{\partial t} = -\frac{\Delta n_{\mathrm{e}}}{\tau_{\mathrm{e}}} - \mu_{\mathrm{e}}\Delta n_{\mathrm{e}}\frac{\partial E}{\partial x} + \mu_{\mathrm{e}}E\frac{\partial \Delta n_{\mathrm{e}}}{\partial x} + D_{\mathrm{e}}\frac{\partial^2 \Delta n_{\mathrm{e}}}{\partial x^2}. \tag{8}$$

Solving Supplementary Equation (8) for the steady-state $\partial n_{\mathrm{e}}/\partial t = 0$, we obtain:

$$\frac{\partial^2 \Delta n_{\mathrm{e}}}{\partial x^2} + \frac{\mu_{\mathrm{e}}}{D_{\mathrm{e}}}E(x)\frac{\partial \Delta n_{\mathrm{e}}}{\partial x} - \left(\frac{1}{\tau_{\mathrm{e}}D_{\mathrm{e}}} + \frac{\mu_{\mathrm{e}}}{D_{\mathrm{e}}}\frac{\partial E(x)}{\partial x}\right)\Delta n_{\mathrm{e}} = 0. \tag{9}$$

The solution of Supplementary Equation (9) allows to know Δn_{e} in each point away from the illumination and, therefore, the current can be calculated as:

$$I = -q\int_0^L \left(n_\mathrm{e}(x)\mu_\mathrm{e}E(x) + D_\mathrm{e}\frac{\partial\Delta n_\mathrm{e}(x)}{\partial x}\right)dx, \tag{10}$$

where the integral is carried out along the length of the device L.

In order to solve Supplementary Equation (9) the electric field distribution $E(x)$ is required. Depending on the band structure and the energy profile of photoexcited particles, three type of funnelling mechanisms are possible[12]: (1) Type I funnel, where the energy level of electrons continuously decreases towards the region of strain, while that of holes increases; (2) Type II funnel, where both levels decrease with increasing strain and the exciton binding energy is small (weakly bound excitons); (3) Type III funnel, in which the behaviour of the bandgap is the same as in type II but in the presence of strongly bound excitons. From the bandgap profiles shown in Figure 1b of the main text, and from the absence of a strong excitonic peak in the PL spectrum of HfS2 (see Supplementary Figure 5), we conclude that our device is a Type II funnel. In this case, due to the weak exciton binding energy, it is possible to replace the potential gradient generated by the strain-induced bandgap modulation with and effective electric field. The simplest model we can adopt is a linear potential from the strained interface (x_j) across a region of length l_j, as depicted schematically in Supplementary Figure 10. The corresponding electric field $E = -\boldsymbol{\nabla}V$ is given by:

$$E(x) = \begin{cases} -\frac{V_\mathrm{sd}}{L} \pm \frac{V_0}{l_\mathrm{j}}, & x_\mathrm{j} - \frac{l_\mathrm{j}}{2} \leq x \leq x_\mathrm{j} + \frac{l_\mathrm{j}}{2} \\ -\frac{V_\mathrm{sd}}{L}, & \text{otherwise} \end{cases} = \begin{cases} -E_\mathrm{sd} \pm E_0, & x_\mathrm{j} - \frac{l_\mathrm{j}}{2} \leq x \leq x_\mathrm{j} + \frac{l_\mathrm{j}}{2} \\ -E_\mathrm{sd}, & \text{otherwise} \end{cases}, \tag{11}$$

which represents the superposition of the applied electric field E_sd and the built-in field due to the strain-induced bandgap modulation E_0. The plus (minus) sign applies to the left (right) junction, respectively. It is important to notice that the sign of E_sd depends on the applied bias, whilst the sign of E_0 depends on the bandgap gradient and it is fixed at each strain junction. Substituting Supplementary Equation (11) in Supplementary Equation (10) we obtain:

$$\Delta n_\mathrm{e} = \begin{cases} \Delta n_\mathrm{e}^0 \exp\left\{ -\frac{1}{2}\left(\frac{q}{k_\mathrm{b}T}(Esd \pm E_0) + \sqrt{\left(\frac{q}{k_\mathrm{b}T}\left(E_\mathrm{sd} \pm E_0\right)\right)^2 + \frac{4}{\tau_\mathrm{e}D_\mathrm{e}}} \right)|x - x_0| \right\}, & x_\mathrm{j} - \frac{l_\mathrm{j}}{2} \leq x \leq x_\mathrm{j} + \frac{l_\mathrm{j}}{2} \\ \Delta n_\mathrm{e}^0 \exp\left\{ -\frac{1}{2}\left(\frac{q}{k_\mathrm{b}T}E_\mathrm{sd} + \sqrt{\left(\frac{q}{k_\mathrm{b}T}\right)^2 E_\mathrm{sd}^2 + \frac{4}{\tau_\mathrm{e}D_\mathrm{e}}} \right)|x - x_0| \right\}, & \text{otherwise} \end{cases}, \tag{12}$$

where Δn_e^0 is the excited carrier density at the injection (illumination) point $x = x_0$. We can therefore use Supplementary Equation (12) and Supplementary Equation (10) to simulate an SPCM experiment by calculating the total charge injected in the channel at each injection point $0 \leq x_0 \leq L$, taking into account that in the oxide region ($x_\mathrm{ox} - \frac{l_\mathrm{ox}}{2} \leq x \leq x_\mathrm{ox} + \frac{l_\mathrm{ox}}{2}$, see Supplementary Figure 10) $\Delta n_\mathrm{e}^0 = 0$, since the bandgap of the oxide is greater than the energy of the impinging photon.

The fit shown in Figure 4f, main text, is performed using a value of mobility of $\mu_e = 2.4\,\mathrm{cm^2V^{-1}s^{-1}}$, which is typical for these devices[13]. The injected charges can be estimated from the photon flux $\phi = P_\mathrm{laser}/E_\mathrm{laser} \simeq 3.5\cdot10^{20}\,\mathrm{s^{-1}cm^{-2}}$ and the internal quantum efficiency $\eta_i = (I_\mathrm{ph}E_\mathrm{laser}/qP_\mathrm{laser})/(1 - R - T) \simeq 3.0\cdot10^{-4}$ (where R and T are the reflectivity and transmittance, respectively, as shown in Supplementary Figure 5b), which give a value of injected carriers $\Delta n_\mathrm{e}^0 = 1.05\cdot10^{17}\,\mathrm{s^{-1}cm^{-2}}$. The value of the built-in potential is taken as the difference between the conduction band minimum (CBm) of the strained and unstrained semiconductor, as shown in Supplementary Figure 7c-d, and it is equal to $V_0 = 0.24\,\mathrm{V}$. The

length of the channel is $L = 26\,\mu$m and $T = 300\,$K. The position of the strained junctions can be determined from Figure 2, main text: $l_{\rm j1} = 2.5\,\mu$m (left) and $l_{\rm j2} = 2.0\,\mu$m (right) while the oxide region is $x_{\rm ox} = 15.0\,\mu$m and $l_{\rm ox} = 1.0\,\mu$m. The carriers lifetime, dominated by non-radiative recombination, is left as a free parameter since, to the best of our knowledge, no experiments are reported in literature with measurement of this value for HfS_2. We find that to obtain a good fit and reproduce qualitatively the experimental data we have to assume two different relaxation times for the strained (τ_1) and unstrained (τ_2) regions. We find $\tau_1 \simeq 1 \cdot 10^{-6}\,$s and $\tau_2 \simeq 1 \cdot 10^{-12}\,$s. The two different values could be explained by a change in electron-hole binding energy due to strain, as previously suggested for different materials[14].

Supplementary Discussion

PV enhancement using charge funnelling

The main losses in a photovoltaic device are given by two factors: (1) lack of absorption of photons with energy E smaller than the bandgap of the photoactive material E_g and (2) dissipation of the kinetic energy of carriers with energy $E \gg E_\mathrm{g}$. The first process accounts for 23 % of the energy of the sun whilst the second for 33 %[15]. Charge funnelling can tackle both problems. A solution to the first problem has been proposed based on multiple-bandgap tandem solar-cells. With this respect, funnelling offers the ability to continuously tune the bandgap of the active material, effectively realizing a continuous tandem solar cell. The second factor is related to carrier dynamics: after photon absorption carriers are excited in a distribution which mimics the energy distribution of the photons. After a few hundred femtoseconds, carrier-carrier scattering equilibrates this distribution to a population which can be described by a temperature T_H. In the following tens of picoseconds, carriers loose kinetic energy via phonon emission and relax to a distribution in quasi-equilibrium with the lattice temperature T_a, a process known as cooling. After this, carriers recombine in different ways to return the system to equilibrium. This dynamics is taken into account in estimating the Shockley-Queisser[16]limit by considering that carriers are completely cooled when extracted at temperature T_a, for a given bandgap. This calculation gives a theoretical limit of $\sim 31\,\%$ efficiency for a bandgap of $E_\mathrm{g} \sim 1.3\,\mathrm{eV}$, under the illumination of a blackbody equivalent to 1 Sun at 5760 K.

As we show in our work, inverse charge funnelling reduces the recombination lifetime of photoexcited carriers from $10^{-10}\,\mathrm{s}$ to $10^{-6}\,\mathrm{s}$. Furthermore, thanks to the modulation of the bandgap and the consequent built-in field in the strain region, the drift velocity is expected to increase. The cooling rate of electrons and holes is inversely proportional to the relaxation time whilst the recombination lifetime is proportional to it[17]. Therefore, the observed reduction of the recombination lifetime can be related to the slowing of the cooling process, which, together with the increased drift velocity, allow the distribution of hot carriers to be maintained at a temperature T_h, where $T_\mathrm{H} > T_\mathrm{h} > T_\mathrm{a}$. The exploitation of hot-carriers extraction has been largely investigated and the maximum efficiency of a photovoltaic device can be theoretically estimated[15] to be $\sim 65\,\%$ at $E_\mathrm{g} \sim 0.5\,\mathrm{eV}$, under the illumination of a blackbody equivalent to 1 Sun at 5760 K, for a carrier temperature of 3600 K. It is possible to compute such limit for the case of HfS_2. The estimation of the maximum efficiency of a hot-carrier solar cells relies on a number of assumptions, which should carefully be reviewed in light of what is achievable in a funnel device. One of the main assumptions is that the extraction of carrier is performed with selective contacts which have an energy separation between holes and electrons larger than the bandgap ($E_\mathrm{out} > E_\mathrm{g}$, ideally close to the median energy of the hot-electrons distribution) and that extraction is performed in an extremely narrow energy range ($\Delta E_\mathrm{out} \ll k_\mathrm{b}T$). The realization of such effect has been the subject of many studies and attempts to realise hot-carrier extraction using, for example, quantum-confined structures have been reported [18, 19]. By taking the above discussion into consideration, it is possible to calculate the current extracted from a hot carrier solar cell:

$$J \cdot E_\mathrm{out} = q\left[f_\mathrm{s} L(E_\mathrm{g}, \mathrm{inf}, T_\mathrm{a}, 0) - L(E_\mathrm{g}, \mathrm{inf}, T_\mathrm{h}, \mu_\mathrm{h}) + (1 - f_\mathrm{s}) L(E_\mathrm{g}, \mathrm{inf}, T_\mathrm{a}, 0)\right], \qquad (13)$$

where J is the current density, $f_\mathrm{s} = 2.16 \cdot 10^{-5}$ is the angular range of the sun, q is the charge of the electron, μ_h is the chemical potential of the hot electrons and $L(E_\mathrm{min}, E_\mathrm{max}, T, \mu)$ rep-

resents the net emitted or absorbed energy flux density integrated over the range $[E_{\mathrm{min}}, E_{\mathrm{max}}]$ and it is equal to

$$L(E_{\mathrm{min}}, E_{\mathrm{max}}, T, \mu) = \frac{2\pi}{h^3 c^2} \int_{E_{\mathrm{min}}}^{E_{\mathrm{max}}} \frac{E^3}{e^{\frac{E-\mu}{k_{\mathrm{b}}T}} - 1} dE, \tag{14}$$

where h is the Planks constant, c the speed of light and k_B the Boltzmanns constant. The first term in Supplementary Equation (13) represents the energy flux density received by the sun, the second term represents the energy flux density absorbed by the solar cell and the third term represents the energy flux density emitted by the solar cell into the environment (detailed balance limit). From the $J(V)$ curve it is possible to calculate the extracted power $P(V) = V \cdot J$ from the solar cell and, consequently, the maximum efficiency given the three design parameters E_{g}, E_{out} and V. Our calculation shows that, for a carrier temperature of 2000 K it is possible to achieve a maximum efficiency of $\sim 45\,\%$ for a bandgap of 1.96 eV (HfS_2), assuming extraction at the contacts with energy ~ 0.2 eV above the bandgap. Such electron temperature has indeed be reported in graphene[20] and the required type of contacts could be realised, for example, using heterostructures of 2D materials in order to achieve the desired band-alignment[21]. A smaller bandgap can give a higher efficiency, up to 65 %. Such requirement can be easily satisfied in a funnel device owing to the ability of TMDs to sustain levels of strain up to 11 %[22] and the strong layer-dependent bandgap, which has been demonstrated in many 2D materials[23, 24].

Supplementary Figures

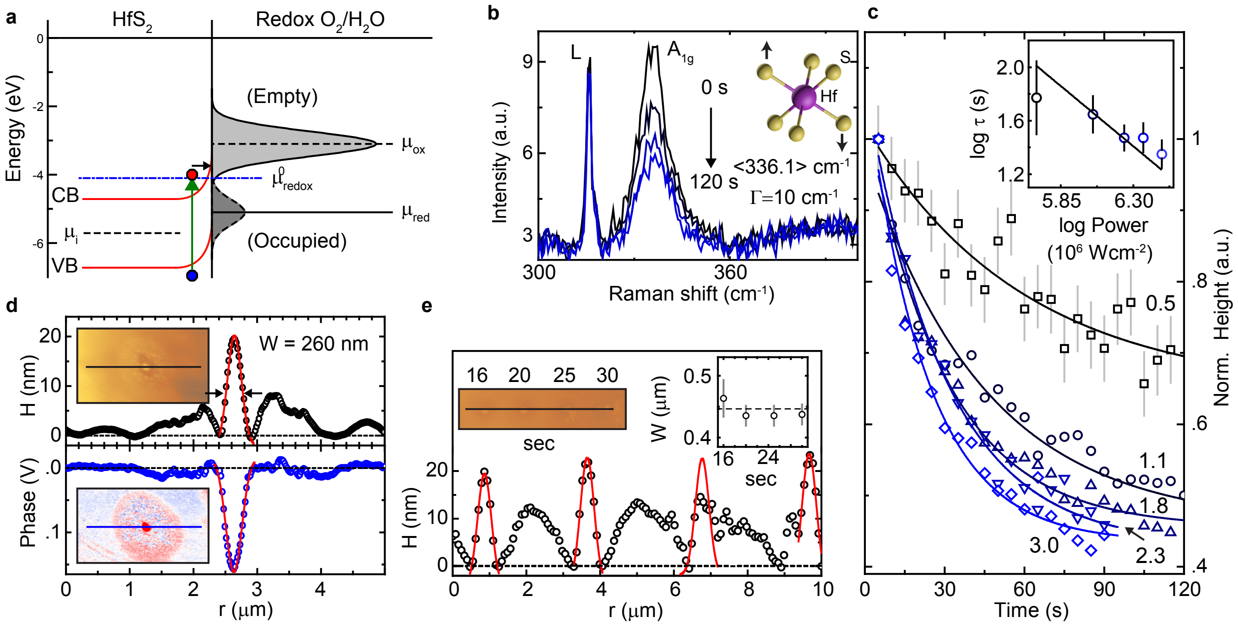

Supplementary Figure 1: **Charge-Transfer model and photo-oxidation of HfS_2.** **a**, Schematic energy diagram of multi-layer HfS_2 in contact with the redox couple O_2/H_2O present in air in atmospheric conditions[2]. The chemical potentials of the oxidizing and reducing species are $\mu_{ox} = -3.1\,eV$ and $\mu_{ox} = -5.1\,eV$, respectively. The chemical potential of the oxygen acceptor state is $\mu^0_{redox} = -4.1\,eV$. The intrinsic work function of HfS_2 is $\mu_i = -5.7\,eV$ with $E_g = 1.96\,eV$. **b**, Time evolution of the HfS_2 A_{1g} Raman mode at $336.1 \pm 0.01\,cm^{-1}$ (average), with FWHM $10\,cm^{-1}$, upon laser exposure ($\lambda = 514\,nm$, P = $3.0\,MW\,cm^{-2}$) of a 4.9 nm thick flake. The peak named L corresponds to a spurious laser line (note no change of this line in the spectra). **c**, Normalized A_{1g} mode height as a function of time for different incident laser powers ($0.5-3.0\,MW\,cm^{-2}$), solid lines mark monoexponential decays; Inset: log-log plot of decay time τ versus incident power density, solid line marks a slope of -1. **d**, AFM topography (top) and phase signal (bottom) of a 80 nm-thick laser-irradiated flake ($\lambda = 375\,nm$, P = $1.5\,MW\,cm^{-2}$, 10 seconds exposure), a Gaussian peak can be fitted with a FWHM of 260 nm. **e**, AFM topography of the same flake with laser-irradiated spots for different exposure times ($\lambda = 473\,nm$, P = $4.3\,MW\,cm^{-2}$). Inset: FWHM of the Gaussian fits as a function of exposure time, dashed line marks the average value of 446 nm. Error bars represent the uncertainty of the Lorentzian fit of the spectra.

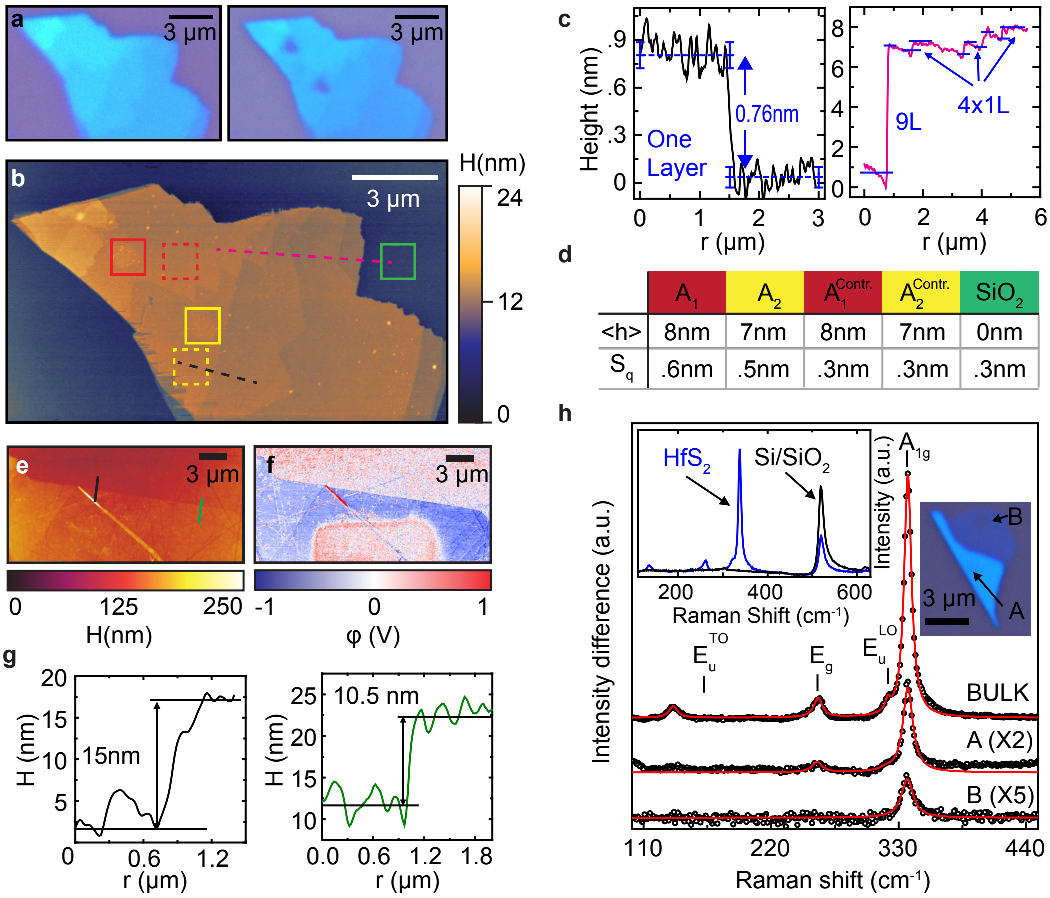

Supplementary Figure 2: **Supplementary AFM data on laser-induced oxidation.** **a**, Optical micrographs of an HfS_2 flake before (left) and after (rigth) exposure of two areas with $\lambda_{exc} = 532\,\mathrm{nm}$ at $0.1\,\mathrm{MW\,cm^{-2}}$ incident power for 20 s. **b**, AFM topography of the same flake shown in **a**. The straight dashed lines correspond to the regions for profiles shown in panel **c**, where the height of a single layer is measured to be $0.76 \pm 0.08\,\mathrm{nm}$. Solid squares enclose the laser-exposed regions, dashed squares enclose the control regions used for statistical analysis. **d**, Statistical analysis of AFM topography shown in **b**: $< \mathrm{h} >$ is the average height measured in the square and S_q is the root-mean-square (rms) surface roughness. **e**, AFM topography and **f** tapping phase images of the HfS_2 flake presented in Figure 1a main text, after laser exposure of the central area. **g**, Height profiles across the solid lines shown in **e**. **h**, Raman spectra of HfS_2 acquired on a bulk sample ($> 100\,\mu$m thick) and on a thinner flake exfoliated on Si/SiO_2 (areas A and B in the optical micrograph), after subtraction of the pristine substrate spectrum (inset).

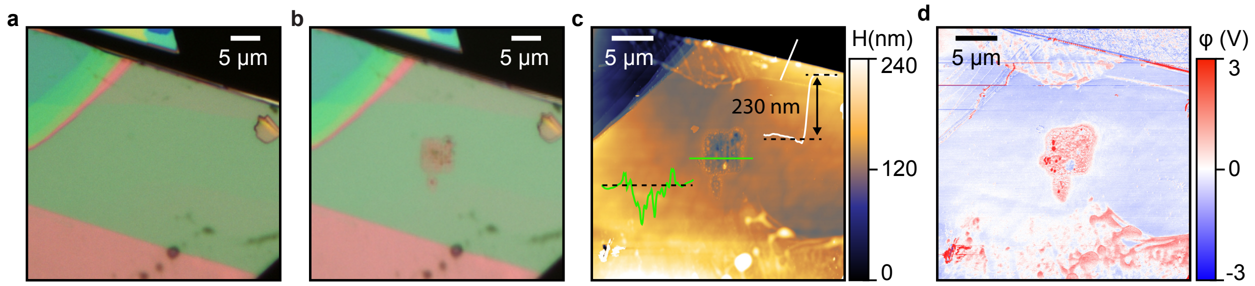

Supplementary Figure 3: **Thick flakes photo-oxidation and damage.** **a-b**, Optical micrograph of a thick flake before (**a**) and after (**b**) laser exposure of a 5×5 μm square, with $\lambda_{\rm exc} = 375$ nm ($P = 5.0$ MW/cm^2), scanned in steps of 0.5 μm, exposing each point for 20 s. **c-d**, AFM topography (**c**) and tapping phase (**d**) images of the flake shown in **a**. Insets show the thickness of the flake (white), $\delta = 230$ nm, and the height profile of the laser exposed area (green), the black dotted line marks the average height of the pristine flake.

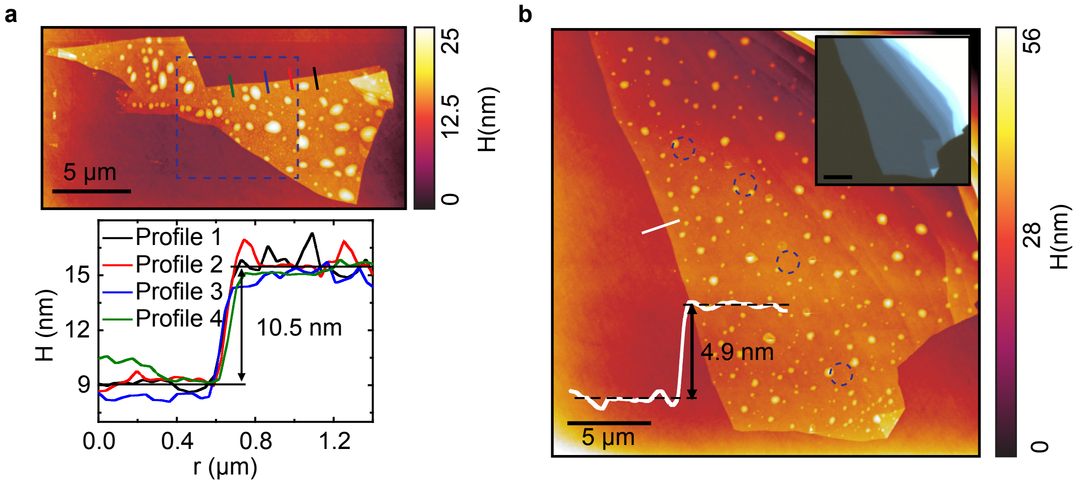

Supplementary Figure 4: **Absorption coefficient and photooxidation AFM data.** **a**, AFM topography (top) of the HfS_2 flake presented in Figure 3a, main text, after laser exposure of the area marked by the dotted square (see main text). Height profiles across the solid lines are shown in the bottom panel: an average flake thickness of 10.5 nm is reported, corresponding to $\sim$ 14 layers. **b**, AFM topography of the flake used in the photooxidation study shown in Figure 2, main text. Dashed circles mark the position where the Raman signal was acquired as a function of time. The flake thickness is 4.9 nm, corresponding to $\sim$ 7 layers. Inset: optical micrograph of the flake immediately after exfoliation on Quartz substrate.

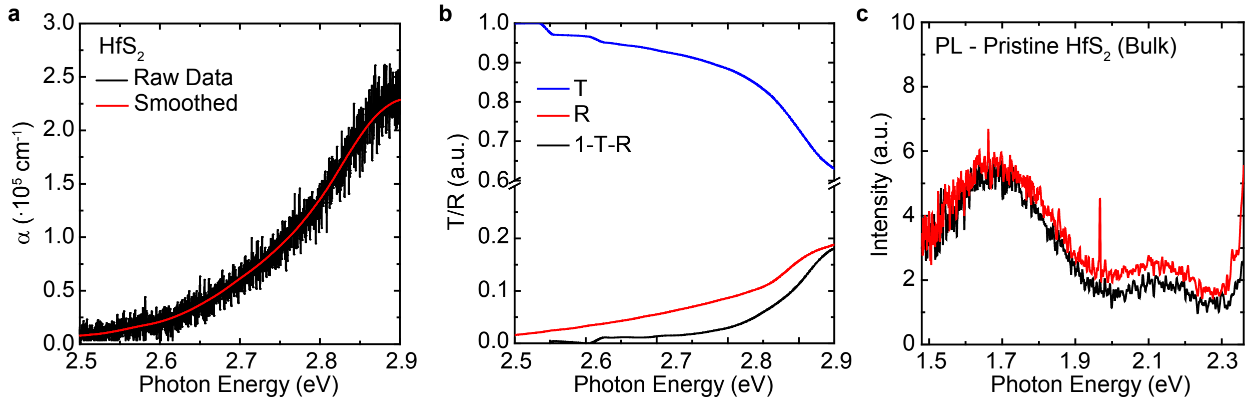

Supplementary Figure 5: **Absorption coefficient α of pristine HfS_2. a**, α as a function of photon energy in the visible range, as shown in Figure 4a, main text. Raw (black) and smoothed (red) data are presented. **b**, Normalised transmittance (T), reflectance (R) and absorbance (1-T-R) as a function of photon energy. **c**, Photoluminescence (PL) spectra of two representative flakes of bulk HfS_2 under 473 nm excitation.

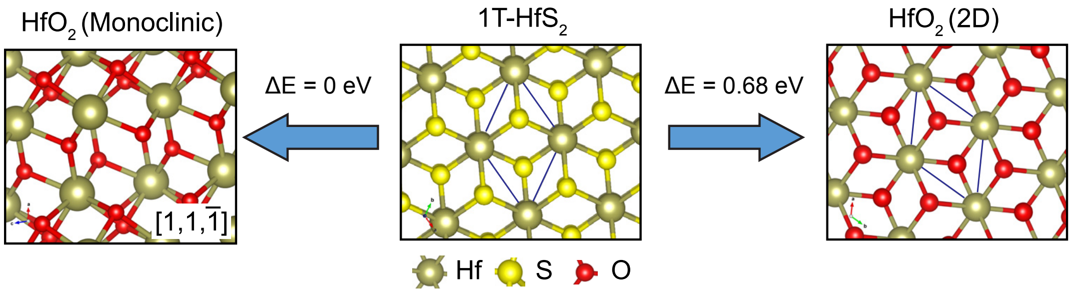

Supplementary Figure 6: **Reactions energy cost and crystal structures** Simulated crystal structures and energy costs of the proposed reactions. The energy difference ΔE is calculated with respect to the formation of monoclinic HfO_2 (E = −11.58 eV). Unit cell highlighted in blue.

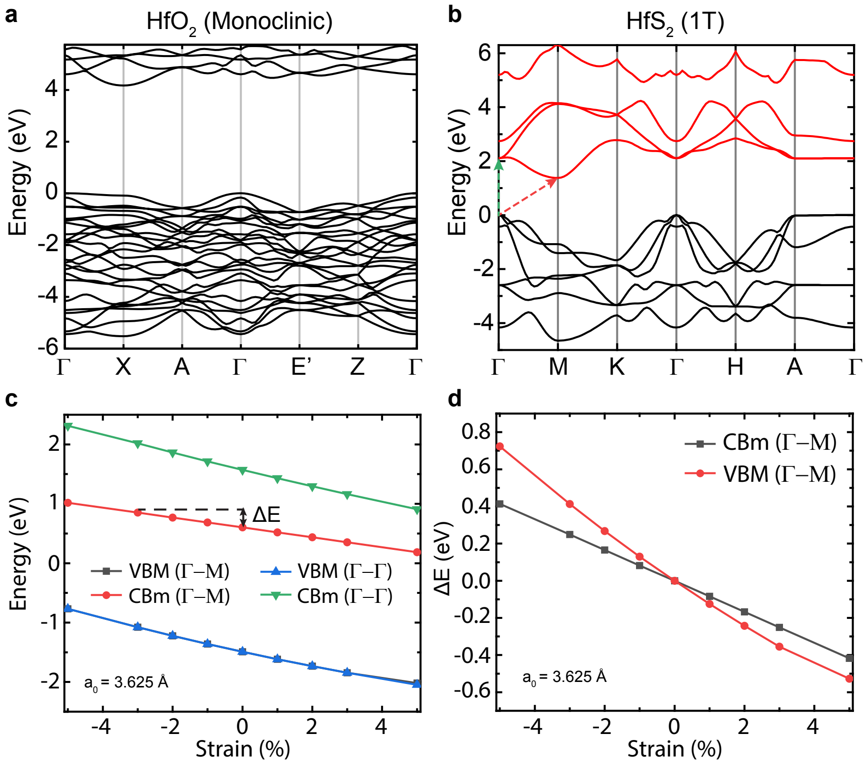

Supplementary Figure 7: **Band structures of examined compounds.** **a**, Calculated band structure of monoclinic HfO_2. **b**, Calculated band structure of 1T-HfS_2. The energy scale is relative to the highest occupied state (0 eV). Black (red) are valence (conduction) states. **c**, Conduction band minimum (CBm) and valence band maximum (VBM) in the $\Gamma - \Gamma$ direction (red arrow in panel **b**) and in the $\Gamma - \mathrm{M}$ direction (green arrow in panel **b**), as a function of strain. **d**, Difference between the unstrained and strained VBM and CBm in the $\Gamma - \mathrm{M}$ direction.

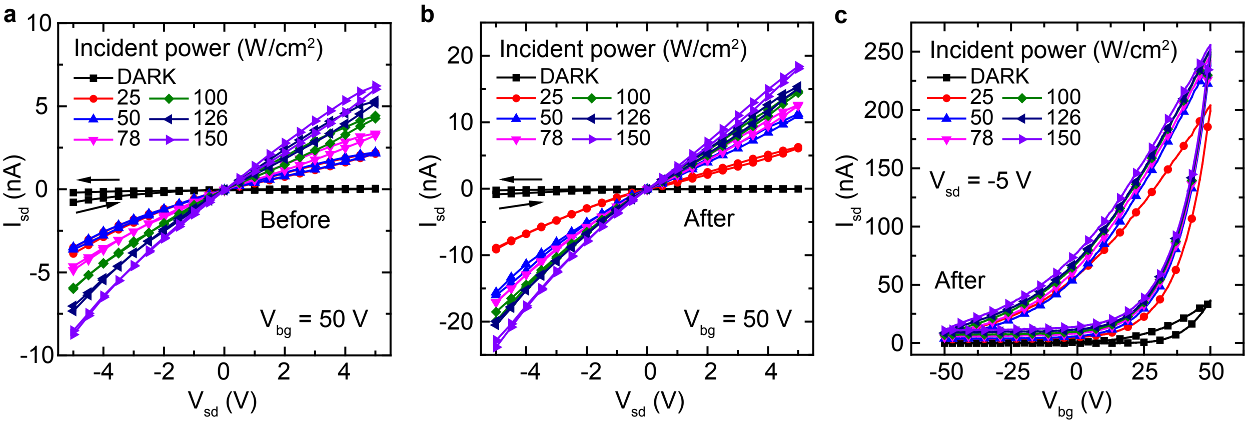

Supplementary Figure 8: **Electrical characteristic of HfS_2 devices.** **a**, Current-voltage (I_{sd}-V_{sd}) characteristic of the device shown in Figure 3 main text, before laser-assisted oxidation in the dark and for different incident optical powers ($\lambda = 473$ nm, $V_{bg} = +50$ V). **b**, Current-voltage characteristic of the same device after laser-assisted oxidation. **c**, Gate voltage dependence of the photocurrent for the same device, after laser-assisted oxidation, $V_{sd} = -5$ V.

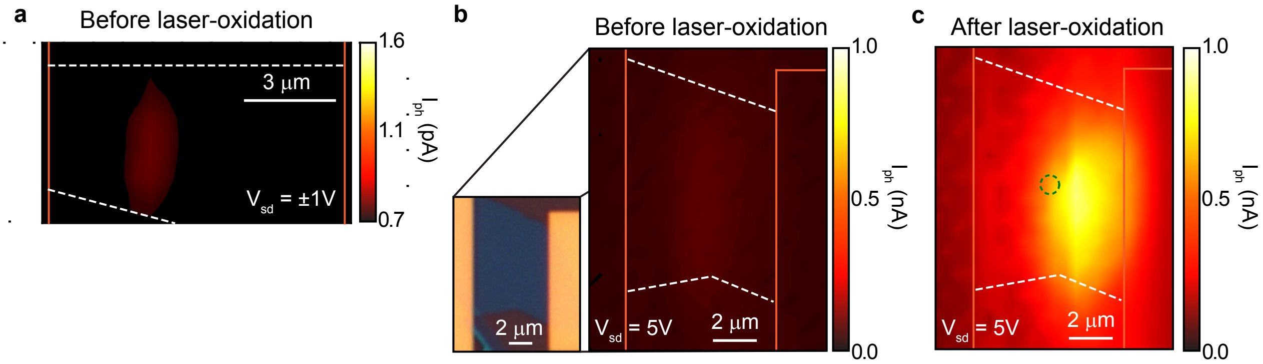

Supplementary Figure 9: **Extended photocurrent data of HfS_2 devices.** **a**, Scanning photocurrent microscopy map (SPCM) of the device in Figure 4, main text, before laser assisted oxidation. Under $V_{sd} = \pm 1\,\mathrm{V}$ only a small photocurrent is observed. For $V_{sd} = 0\,\mathrm{V}$ no photoresponse could be measured at the sensitivity of our instruments. **b-c**, SPCM maps of another device before and after (**c**) laser assisted oxidation in the centre of the flake (green circle), under $V_{sd} = 5\,\mathrm{V}$. Optical micrograph of the device is shown in panel **b**, inset. All SPCM measurements were performed using $\lambda = 473$ nm at a power $P = 150$ W/cm^2, as in Figure 4, main text.

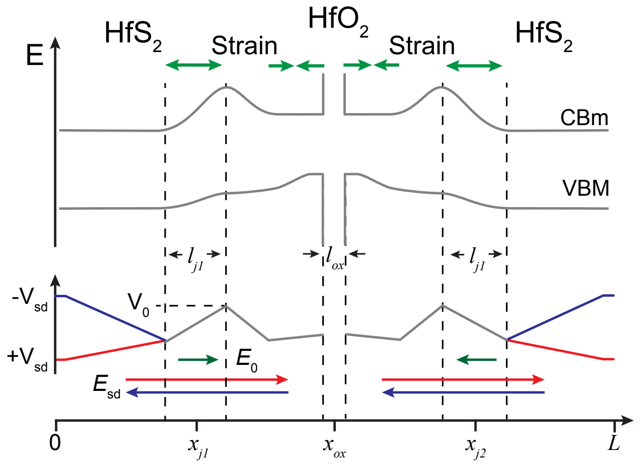

Supplementary Figure 10: **SPCM model in a strained device.** Schematic band diagram of a strained device with the oxide region at the centre of the channel. There are four strain regions (green arrows), two compressive in the vicinity of the oxide and two tensile far away from it. The simplified potential is shown below for positive ($+V_{\mathrm{sd}}$) and negative ($-V_{\mathrm{sd}}$) bias.

Supplementary Tables

Supplementary Table 1: Reactions energetics

Reaction	Energy cost (eV)	Energy cost (eV) per HfS_2	ΔE (eV)[a] per HfS_2
$HfS_2 + 3\,O_2 \longrightarrow HfO_2 + 2\,SO_2$[b]	-11.58	-11.58	
$32\,HfS_2 + (32 + 64)\,O_2 \longrightarrow Hf_{32}O_{64} + 64\,SO_2$[c]	-370.55	-11.58	
$HfS_2 + 3\,O_2 \longrightarrow HfO_2(2\,D) + 2\,SO_2$[f]	-10.90	-10.90	0.68
$HfS_2 + 3\,O_2 \longrightarrow HfO_2(cubic) + 2\,SO_2$[g]	-11.28	-11.28	0.30

[a]Compared to lowest energy reaction; [b]$1 \times 1 \times 1$ Primary unit cell of monoclinic HfO_2; [c]Large $2 \times 2 \times 2$ unit cell of monoclinic HfO_2; [f]Energy cost to produce 2D HfO_2; [g]Energy cost to produce cubic HfO_2;

Supplementary References

[1] Favron, A. *et al.* Photooxidation and quantum confinement effects in exfoliated black phosphorus. *Nat Mater* **14**, 826–832 (2015).

[2] Memming, R. *Semiconductor Electrochemistry* (Wiley, 2008).

[3] Iwasaki, T., Kuroda, N. & Nishina, Y. Anisotropy of lattice dynamical properties in zrs_2 and hfs_2. *Journal of the Physical Society of Japan* **51**, 2233–2240 (1982).

[4] Cingolani, A., Lugar, M., Scamarcio, G. & Lvy, F. The raman scattering in hafnium disulfide. *Solid State Communications* **62**, 121 – 123 (1987).

[5] Roubi, L. & Carlone, C. Resonance raman spectrum of hfs_2 and zrs_2. *Phys. Rev. B* **37**, 6808–6812 (1988).

[6] Greenaway, D. & Nitsche, R. Preparation and optical properties of group iv-vi chalcogenides having the cdi_2 structure. *Journal of Physics and Chemistry of Solids* **26**, 1445 – 1458 (1965).

[7] Nath, M. & Rao, C. N. R. Nanotubes of group 4 metal disulfides. *Angewandte Chemie International Edition* **41**, 3451–3454 (2002).

[8] Traving, M. *et al.* Combined photoemission and inverse photoemission study of hfs_2. *Phys. Rev. B* **63**, 035107 (2001).

[9] Rasmussen, F. A. & Thygesen, K. S. Computational 2d materials database: Electronic structure of transition-metal dichalcogenides and oxides. *The Journal of Physical Chemistry C* **119**, 13169–13183 (2015).

[10] Chae, S. H. *et al.* Oxidation effect in octahedral hafnium disulfide thin film. *ACS Nano* **10**, 1309–1316 (2016).

[11] Graham, R. & Yu, D. Scanning Photocurrent Microscopy in Semiconductor Nanostructures. *Modern Physics Letters B* **27**, 1330018 (2013).

[12] Feng, J., Qian, X., Huang, C.-W. & Li, J. Strain-engineered artificial atom as a broad-spectrum solar energy funnel. *Nat Photon* **6**, 866–872 (2012).

[13] Xu, K. *et al.* Ultrasensitive phototransistors based on few-layered hfs_2. *Advanced Materials* **27**, 7881–7887 (2015).

[14] San-Jose, P., Parente, V., Guinea, F., Roldán, R. & Prada, E. Inverse funnel effect of excitons in strained black phosphorus. *Physical Review X* **6**, 1–12 (2016).

[15] Nelson, J. *The Physics of Solar Cells*, chap. 10, 289–325 (Imperial College Press, 2011), 1st edn.

[16] Shockley, W. & Queisser, H. J. Detailed balance limit of efficiency of p-n junction solar cells. *Journal of Applied Physics* **32**, 510–519 (1961).

[17] Sánchez, M. Carrier heating or cooling in semiconductor devices. *Solid-State Electronics* **16**, 549 – 557 (1973).

[18] Nozik, A. J. Spectroscopy and hot electron relaxation dynamics in semiconductor quantum wells and quantum dots. *Annual Review of Physical Chemistry* **52**, 193–231 (2001).

[19] Watanabe, D., Kasamatsu, N., Harada, Y. & Kita, T. Hot-carrier solar cells using low-dimensional quantum structures. *Applied Physics Letters* **105**, 171904 (2014).

[20] Johannsen, J. C. *et al.* Direct view of hot carrier dynamics in graphene. *Phys. Rev. Lett.* **111**, 027403 (2013).

[21] Özçelik, V. O., Azadani, J. G., Yang, C., Koester, S. J. & Low, T. Band alignment of two-dimensional semiconductors for designing heterostructures with momentum space matching. *Phys. Rev. B* **94**, 035125 (2016).

[22] Bertolazzi, S., Brivio, J. & Kis, A. Stretching and breaking of ultrathin mos2. *ACS Nano* **5**, 9703–9709 (2011).

[23] Xia, F., Wang, H. & Jia, Y. Rediscovering black phosphorus as an anisotropic layered material for optoelectronics and electronics. *Nature Communications* **5**, 4458 (2014).

[24] Wang, Q. H., Kalantar-Zadeh, K., Kis, A., Coleman, J. N. & Strano, M. S. Electronics and optoelectronics of two-dimensional transition metal dichalcogenides. *Nat Nano* **7**, 699–712 (2012).
